# Effect of water temperature and population density on the population dynamics of *Schistosoma mansoni* intermediate host snails

**DOI:** 10.1186/s13071-014-0503-9

**Published:** 2014-11-12

**Authors:** Nicky McCreesh, Moses Arinaitwe, Wilber Arineitwe, Edridah M Tukahebwa, Mark Booth

**Affiliations:** School of Medicine, Pharmacy and Health, Durham University, Durham, DH1 3LE UK; Vector Control Division, Ministry of Health, Plot 15 Bombo Road, Kampala, Uganda

**Keywords:** Schistosomiasis, *Schistosoma mansoni*, *Biomphalaria*, Malacology, Climate change, Ecology

## Abstract

**Background:**

Mathematical models can be used to identify areas at risk of increased or new schistosomiasis transmission as a result of climate change. The results of these models can be very different when parameterised to different species of host snail, which have varying temperature preferences. Currently, the experimental data needed by these models are available for only a few species of snail. The choice of density-dependent functions can also affect model results, but the effects of increasing densities on *Biomphalaria* populations have only previously been investigated in artificial aquariums.

**Methods:**

Laboratory experiments were conducted to estimate *Biomphalaria sudanica* mortality, fecundity and growth rates at ten different constant water temperatures, ranging from 13-32°C. Snail cages were used to determine the effects of snail densities on *B. sudanica* and *B. stanleyi* mortality and fecundity rates in semi-natural conditions in Lake Albert.

**Results:**

*B. sudanica* survival and fecundity were highest at 20°C and 22°C respectively. Growth in shell diameter was estimated to be highest at 23°C in small and medium sized snails, but the relationship between temperature and growth was not clear. The fecundity of both *B. sudanica* and *B. stanleyi* decreased by 72-75% with a four-fold increase in population density. Increasing densities four-fold also doubled *B. stanleyi* mortality rates, but had no effect on the survival of *B. sudanica*.

**Conclusions:**

The optimum temperature for fecundity was lower for *B. sudanica* than for previously studied species of *Biomphalaria*. In contrast to other *Biomphalaria* species, *B. sudanica* have a distinct peak temperature for survival, as opposed to a plateau of highly suitable temperatures. For both *B. stanleyi* and *B. sudanica*, fecundity decreased with increasing population densities. This means that snail populations may experience large fluctuations in numbers, even in the absence of any external factors such as seasonal temperature changes. Survival also decreased with increasing density for *B. stanleyi*, in contrast to *B. sudanica* and other studied *Biomphalaria* species where only fecundity has been shown to decrease.

**Electronic supplementary material:**

The online version of this article (doi:10.1186/s13071-014-0503-9) contains supplementary material, which is available to authorized users.

## Background

Mathematical models of water temperature and the lifecycles of schistosomes and their intermediate host snails can be used to gain valuable insight into the possible effects of environmental changes, including climate change, on the distribution and intensity of schistosomiasis [1-4]. It is crucial, however, to consider the species of intermediate host snail found in an area when parameterising a model, and when using the model to make predictions of the potential effects of environmental change on schistosome transmission [5,6]. To accurately represent a snail species in these models, it is necessary to have a wide range of data. This includes data on mortality, egg production, egg hatching and juvenile development rates at a range of different temperatures. Experiments determining the effects of other abiotic factors (e.g. the chemical composition of the water) on different life stages of the snails will also advance model development. Currently, sufficient data are only available to accurately parameterise models of temperature and snail ecology to three species of *Biomphalaria*: *B. pfeifferi*, *B. alexandrina*, and *B. glabrata* [6], and (with the exception *B. alexandrina* in South Sudan) only one of these species can be found in sub-Saharan Africa [7], where the majority of schistosomiasis occurs [8]. While *B. pfeifferi* is generally considered to be the most important *S. mansoni* intermediate host in sub-Saharan Africa due to its widespread distribution [9,10], other species are also important in maintaining transmission in many areas. This is particularly the case for lakeside areas in eastern Africa, where prevalences and intensities of intestinal schistosomiasis are often very high [11], and where *B. pfeifferi* are not commonly found in large numbers [9,12-14].

A critical decision in the design of mathematical models of snail population dynamics is how to incorporate density dependent regulatory factors. In the absence of these factors, simulated snail populations can increase exponentially. This is clearly unrealistic, as wild snail populations are limited in number by constraints such as food availability. Density-dependent factors can be incorporated into models in two main ways: by increasing mortality rates and/or by decreasing fecundity as densities increase. Laboratory experiments suggest that the fecundity of *B. pfeifferi*, *B. alexandrina*, and *B. glabrata* tends to drop in unfavourable conditions, with little increase in mortality rates [15-18]. It is unclear if this is the case in more natural environments, or for other species of *Biomphalaria*.

In this paper, we describe laboratory and field experiments conducted with two species of *Biomphalaria*: *B. sudanica* and *B. stanleyi. B. sudanica* is found in lakes and rivers throughout central and eastern Africa, and is particularly common in Uganda [7,9]. *B. stanleyi* has a more limited distribution, and has been found in Lake Chad in north-central Africa, and Lake Albert and Lake Cohoha in eastern Africa. Both are capable of acting as intermediate hosts for *S. mansoni* [9]. The results of our experiments provide crucial data that can be used to inform future model parameterisation. We determine the effects of water temperature on *B. sudanica* mortality, fecundity, and growth rates, and the effects of population density on *B. sudanica* and *B. stanleyi* mortality and fecundity.

## Methods

### Effect of water temperature on *B. sudanica* population dynamics

#### Experimental procedures

Five 60 litre aquariums were filled with ‘aged’ tap water [19]. Chemical and mechanical water filters were used to clean the water, and water pumps were used to oxygenate it. Pumps were set to a speed which did not disturb the snails. Larger particles of waste were removed from the base of the aquariums each week using a section of hose and suction, and the small volumes of water removed were replaced with aged tap water. This procedure, in conjunction with the water filters, kept the aquarium water clean and prevented the need to periodically replace large volumes of water. Additional calcium was added to the water in the form of crushed snail shells contained in a fine mesh bag. Two pieces of expanded polystyrene foam were placed into each aquarium to provide a surface on which the snails could lay eggs.

Wild, adult *B. sudanica* snails were collected from Lake Albert in western Uganda and transported to a laboratory in Kampala. They were kept in aquariums at ambient temperature (approximately 25°C) for a minimum of a week to acclimatise, before being used in experiments. Snails that shed cercariae of any species were discarded. The shell diameters of the remaining snails were measured at the widest point using digital callipers, and the shells of the snails were individually marked using queen bee markers affixed with nail varnish. It has previously been shown that small quantities of nail varnish applied to the shell is not detrimental to the snails [20,21]. The snails were then placed into the five aquariums in equal numbers. The aquariums were kept at ambient temperature for two days to allow the snails to acclimatise, after which the water was gradually heated or cooled to the desired temperature. Snails were fed with dried lettuce and spirulina cyanobacteria, which were added to the water as a powder.

Two sets of experiments were conducted. In the first, heaters and coolers were set to keep the five aquariums at constant temperatures of 15°C, 19°C, 23°C, 27°C, and 31°C, and 91 snails were initially added to each aquarium. In the second, the heaters and coolers were set to 13°C, 17°C, 21°C, 25°C, and 29°C, and 99 snails were added.

The aquariums were checked six days a week, and dead snails removed and recorded. Each week, all snails were removed and individually recorded, before being placed back in the aquariums. This was necessary as dead snails could be missed during the daily checking. Each individual snail was recorded as being dead of presumed natural causes, dead of non-natural causes (crushed, sucked into the pump, or found desiccated outside the water), lost, or still alive. Every two weeks, the snails were measured using the methods described above. All measurements were made by the same person. Weekly, the number of egg masses found in the aquariums were counted, and the number of eggs in a random sample of 20 masses in each aquarium counted. The egg masses were then discarded. The experiments were run for eight weeks each.

#### Analysis

Actual temperatures in the aquariums were calculated as the mean of daily minimum and maximum temperatures, averaged over the study period.

In calculating mortality and fecundity rates, data from the first week were discarded to ensure that the results were not biased by the impact of the stress experienced by the snails during the marking and measuring progress, and during the adjustment to their new aquariums. Data from the second set of experiments in weeks 4-6 were not included in the mortality and fecundity analyses, due to suspected interference with the aquariums by rats (see Results for details).

Weekly mortality rates were estimated using a discrete time survival analysis approach. Data from snails that were considered to have died from non-natural causes or that were lost were censored from the week that they were last observed alive. Best fit quadratic lines were fitted through the mean mortality rates using a least squares method, subject to the constraint that mortality rates at all temperatures must be non-negative. A quadratic relationship was chosen based on the clear non-linear relationship between temperature and mortality rates, and the plausible fit to the data given by a quadratic relationship. This was done both separately for each experiment, and using all of the data points from both experiments.

For each temperature, the rate of egg mass production/snail/week, and the mean number of eggs/egg mass were calculated. The estimated rate of egg production/snail/week was also calculated. For each of the three fecundity variables, beta distribution lines were fitted through the data points using a least squares method, subject to the constraint that rates were non-negative at the highest and lowest temperatures at which egg production was observed to occur in either experiment. As beta distribution functions are defined on the interval [0 1] only, two additional parameters were added to the functions to scale the water temperatures. Beta distributions were chosen as they gave a close fit to the data, and were biologically plausible. Model fitting was done both separately for each experiment, and using all of the data points from both experiments together.

Absolute growth in shell diameter was calculated for each snail and fortnight. The observations were split into three groups based on the shell diameter of the snail at the start of the fortnight: < =8 mm (‘small’), >8 mm & < =10 mm (‘medium’), and >10 mm (‘large’). Observations were discarded if they showed a reduction in size of greater than 0.1 mm, as the most likely explanation for this was that a small amount of shell had broken off from around the mouth of the shell. Smaller observed reductions may have been due to measurement error, and were therefore not excluded (as the corresponding small increases due to measurement errors could not be distinguished from genuine growth, and could therefore not be excluded). For each size and temperature, mean growth was calculated. Individual snails contributed one observation for each fortnight for which they had a start and end measurement. For each snail size category, results from both experiments were combined, and linear and quadratic regression were used to investigate the relationship between water temperature and growth. Likelihood-ratio tests were used to compare the linear and quadratic regression models.

### Effect of population density on *B. sudanica* and *B. stanleyi* population dynamics

Eight snail cages were constructed, based on a design used by C Kariuki [22]. The cages consisted of wooden frames, surrounded by a fine plastic mesh that is typically used for fishing nets. No metal was used in the construction of the cages, as metal can be toxic to snails [23]. Four of the cages measured 50 cm × 40 cm × 30 cm deep, and were used for *B. sudanica* snails. The other four measured 25 cm × 40 cm × 60 cm deep, and were used for *B. stanleyi*, which are commonly found in deeper water [14]. Two pieces of expanded polystyrene foam were placed into each cage to provide a surface on which the snails could lay eggs. The cages were placed in Lake Albert, in locations where the respective snail species could be found, and where the depth of the water allowed the cages to reach close to the bottom of the lake. All four cages for each snail species were placed in the same location. The cages were held in place with approximately 5-10 cm of the cage above the surface of the water, giving the snails access to the water surface to breathe. The cages were left in position for two weeks before any snails were added to give time for algae to grow on the cages.

At the start of the experiment, 400 *B. sudanica* snails were collected from a range of locations in Lake Albert, beside three adjacent villages. Fifty *B. sudanica* were added to each of two cages, 100 to one cage, and 200 to the final cage. This gave snail densities of approximately 1 snail/l, 2 snails/l and 4 snails/l respectively. Three weeks later, when *B. stanleyi* numbers near the three villages had risen and sufficient snails could be collected, this was repeated for *B. stanleyi.*

The cages were visited every three to four days. Each visit, dead snails were removed and the number of snails remaining counted. Missing snails were assumed to have died and decomposed, as there was no way for snails to leave the cages, or for predators to enter. Dead and missing snails were replaced with snails from the lake, maintaining 50, 50, 100, and 200 snails in the four cages. On each visit, the number of egg masses in each cage was also counted, and the number of eggs is a random sample of 30 masses in each cage counted. Egg masses were removed from the cages after counting. The experiment was run for 19 weeks in total for *B. sudanica*, and 16 weeks in total for *B. stanleyi.*

For each observation, the length of time since the last observation was calculated. For each snail species and snail density, mean mortality rates, egg masses/snail/day, eggs/egg mass, and eggs/snail/day were calculated. When calculating eggs and egg masses/snail/day, the denominators were approximated by the means of numbers of snails alive at the beginning and end of the relevant observation period.

## Results

### Effect of water temperature on *B. sudanica* populations dynamics

Mean water temperatures in the 10 aquariums were 13.4°C, 15.7°C, 16.7°C, 18.9°C, 20.9°C, 22.8°C, 26.7°C, 28.3°C, 29.5°C, and 32.0°C. Daily minimum and maximum temperatures in each aquarium varied from the mean by an average of 0.5-1.3°C.

Fifty-six snails died during the experiments due to reasons that could be considered ‘non-natural’: five climbed out of the water and died, eight were accidently crushed during measuring, and 43 died after being sucked into the pumps or filters (the majority on a single occasion when the container housing the pumps and filters fell over). In the first set of experiments, a total of 20 snails were lost. This means that they were noted as being alive at the end of one week, but could not be found at the end of the next or any subsequent weeks. In the second set of experiments, a total of 14 snails were lost in weeks 1-3 and 7-8. In weeks 4-6, a total of 271 snails were lost, equivalent to 34-51% of snails each week. This was believed to be due to predation by rats. A number of rats were trapped, and the number of snails being lost fell to earlier levels in the final two weeks of the experiment.

**Figure 1:**
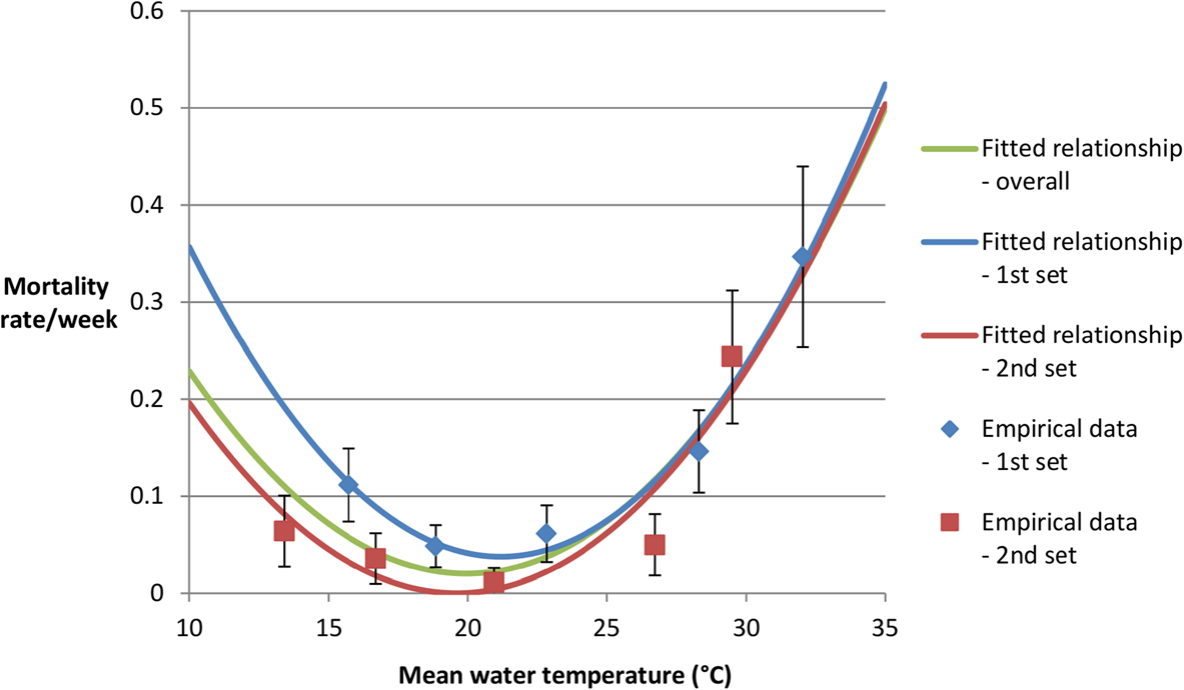
**Relationship between water temperature and snail mortality rates.** Blue diamonds and red squares show daily snail mortality rates calculated from data collected during the 1^st^ and 2^nd^ sets of experiments respectively. Green, blue, and red lines show fitted quadratic relationships between temperature and snail mortality rates, fitted to all data, data collected during the 1^st^ set of experiments only, and data collected during the 2^nd^ set of experiments only respectively.

There was very strong evidence that water temperature had an effect on snail mortality rates, both when all data were considered together and when data from the two sets of experiments were considered separately (p < 0.0001) (Figure 1). Snail survival was estimated to be highest at 19.9°C (Table 1).

**Table 1:** **Optimum temperatures for snail survival and fecundity**

	**Optimum temperature**
**Survival**	19.9°C
**Mean number of eggs/snail/week**	21.6°C
**Mean number of egg masses/snail/week**	21.9°C
**Mean number of eggs/egg mass**	20.4°C
**Mean growth in shell diameter (small snails)**	23.1°C
**Mean growth in shell diameter (medium snails)**	23.3°C
**Mean growth in shell diameter (large snails)**	No evidence for any association.

Optimum temperatures are estimated from equations fitted through empirical data points. Small, medium, and large snails are defined as snails with shell diameters at the start of the fortnight of < =8 mm, >8 mm & < =10 mm. and >10 mm respectively.

**Figure 2:**
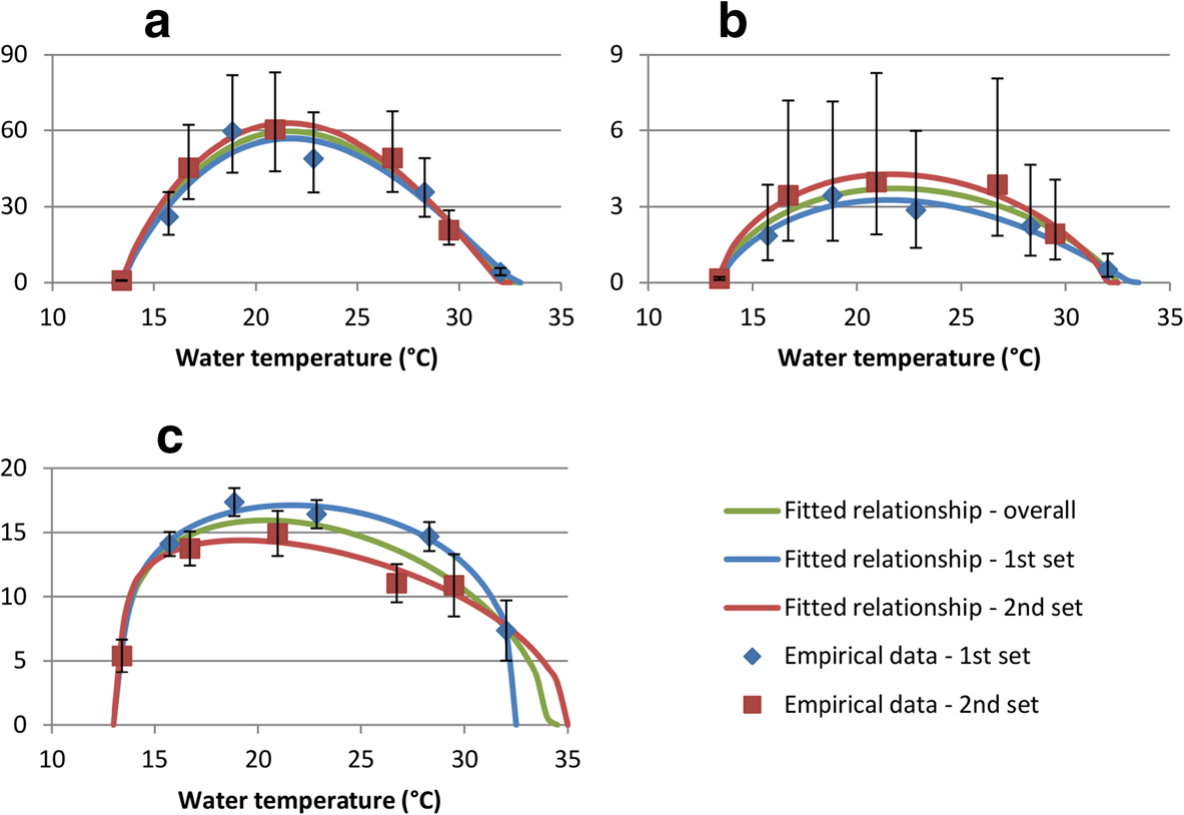
**Relationship between water temperature and snail fecundity. a**) Mean eggs/snail/week. **b**) Mean egg masses/snail/week. **c**) Mean eggs/egg mass. Blue diamonds and red squares show data collected during the 1^st^ and 2^nd^ sets of experiments respectively. Green, blue, and red lines show fitted scaled beta distribution functions, fitted to all data, data collected during the 1^st^ experiment only, and data collected during the 2^nd^ experiment only respectively.

The optimum temperature for snail fecundity (maximum mean number of eggs/snail/week) was estimated to be 21.6°C (Table 1). There was very strong evidence that water temperature had an effect on snail fecundity, both when all data were considered together and when data from the two sets of experiments were considered separately (p < 0.0001) (Figure 2a). This was due both to a reduction in the mean number of egg masses produced/snail, and a reduction in the mean number of eggs/snail mass (Figure 2b and 2c). After taking into account water temperature, there appeared to be little difference between the two sets of experiments in mean egg production (Figure 2a), but the data suggest that snails in the 1^st^ experiments may have produced fewer, larger egg masses, and the snails in the 2^nd^ experiments may have produced more, smaller masses.

**Figure 3:**
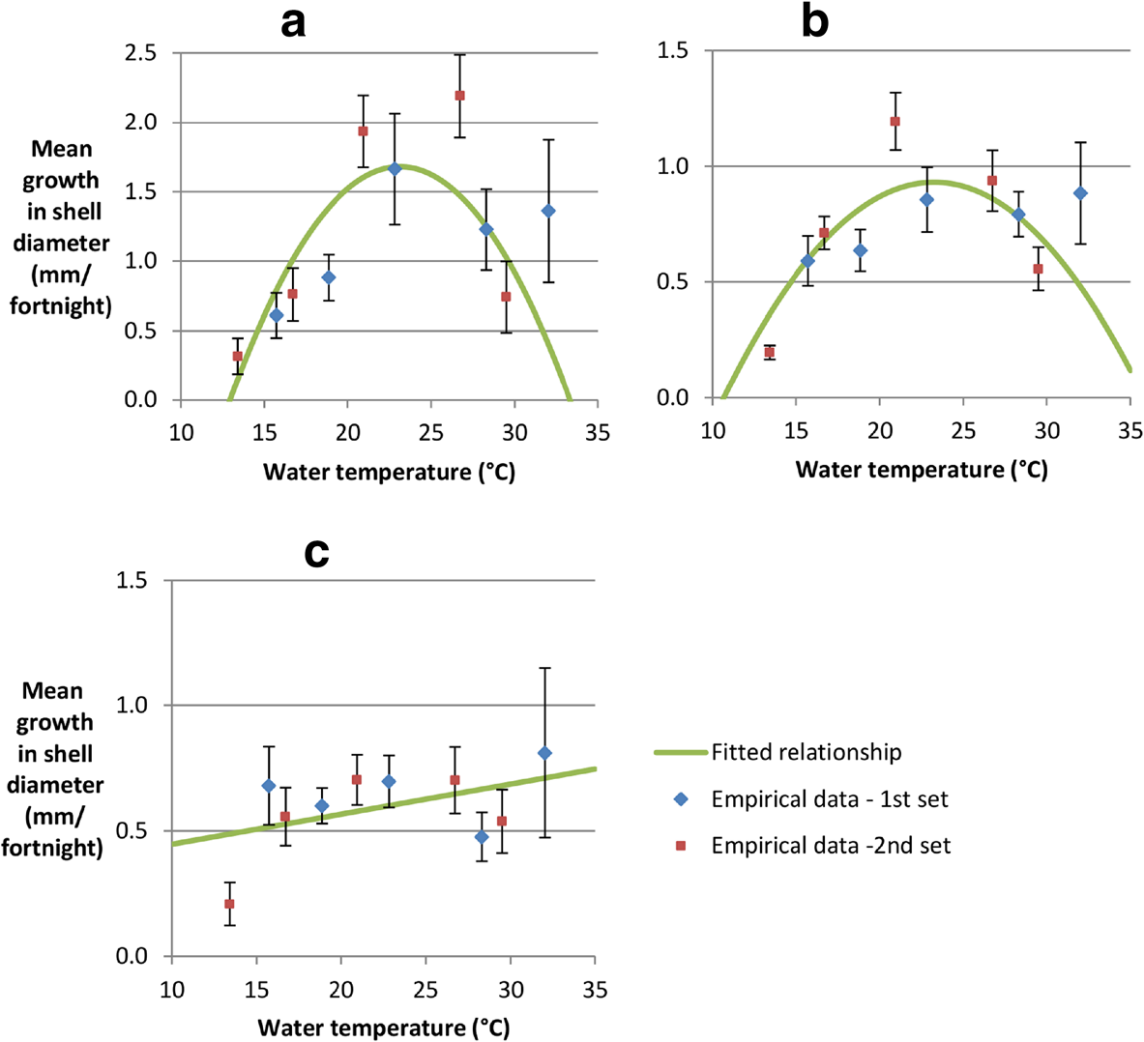
**Relationship between water temperature and snail growth. a**) Small snails. **b**) Medium snails. **c**) Large snails. Small, medium and large snails were defined as snails with a shell diameter at the start of the fortnight of < =8 mm, >8 mm & < =10 mm, and >10 mm respectively. Lines show the best fit linear and quadratic relationships, fitted to data from both sets of experiments combined.

In total, there were 1014 observations on fortnightly growth in shell diameter (141, 498, and 375 in small, medium and large snails respectively). At all temperatures above 13.4°C, mean absolute growth in shell diameter was highest for snails classified as small, and lowest for snails classified as large (Figure 3).

Figure 3 shows mean fortnightly growth in shell diameter by temperature. For small and medium snails, there was very strong evidence that quadratic regression models gave a better fit to the data than linear regression models (p < 0.0001). For large snails, there was no evidence that a quadratic regression model gave a better fit (p = 0.1). Large snails grew by an additional 0.0003 mm only with each 1°C increase in temperature, and the linear trend was not significant (p = 0.99). The fitted linear (large snails) and quadratic (small and medium snails) regression models are shown in Figure 3. The estimated temperatures at which growth in shell diameter was fastest were 23.1°C and 23.3°C for small and medium snails respectively (Table 1). The fitted regression models explained 16%, 11%, and <0.001% of the variation in snail growth for small, medium, and large snails respectively.

Mean mortality, fecundity, and growth rates at each temperature and the fitted equations are shown in Additional file 1.

### Effect of population density on *B. sudanica* and *B. stanleyi* population dynamics

There was very strong evidence of increasing mortality rates with increasing snail densities for *B. stanleyi*, with a mean mortality rate of 0.17/week at the lowest density, 0.23/week at the intermediate density, and 0.35/week and the highest density (p < 0.0001) (Table 2). There was no evidence for any relationship between snail densities and mortality rates for *B. sudanica*.

**Table 2:** **Relationship between snail densities and snail mortality rates and egg production**

	**Snail density (snails/litre)**	**Mortality rate/week**	**Mean egg masses/snail/week**	**Mean eggs/egg mass**	**Mean eggs/snail/week**
		**(95% CI)**	**(95% CI)**	**(95% CI)**	**(95% CI)**
***B. stanleyi***	**1**	0.166 (0.131-0.200)	0.538 (0.473-0.603)	12.2 (11.7-12.8)	6.81 (5.79-7.83)
	**2**	0.232 (0.189-0.276)	0.404 (0.351-0.458)	11.6 (10.9-12.4)	4.90 (4.04-5.76)
	**4**	0.351 (0.300-0.402)	0.183 (0.140-0.227)	9.5 (8.7-10.4)	1.93 (1.39-2.46)
		p < 0.0001	p < 0.0001	p < 0.0001	p < 0.0001
***B. sudanica***	**1**	0.332 (0.258-0.406)	2.54 (2.29-2.79)	17.5 (16.8-18.2)	46.1 (40.5-51.7)
	**2**	0.28 (0.212-0.348)	1.21 (0.97-1.45)	14.4 (13.0-15.8)	19.4 (15.1-23.6)
	**4**	0.308 (0.245-0.371)	0.71 (0.53-0.89)	13.4 (11.8-14.9)	11.4 (7.8-14.9)
		p = 0.6	p < 0.0001	p < 0.0001	p < 0.0001

For both snail species, there was very strong evidence of a decrease in fecundity with increasing snail densities, with *B. sudanica* producing a mean of 46, 19 and 11 eggs/snail/week and *B. stanleyi* producing a mean of 6.8, 4.9 and 1.9 eggs/snail/week at snail densities of 1, 2 and 4 snails/l respectively (p < 0.0001 for both species) (Table 2). This occurred through both a reduction in the mean number of egg masses laid/snail/week at higher population densities, and a reduction in the mean numbers of eggs/egg mass.

## Discussion

### Effect of water temperature on *B. sudanica* populations dynamics

Our experiments demonstrate that water temperature has a substantial effect on *B. sudanica* population dynamics, greatly affecting both mortality and fecundity rates. Our results showed clear, non-linear relationships between water temperature and *B. sudanica* survival and fecundity. Optimum temperatures for both maximum fecundity and survival were low, at only 22°C and 20°C respectively. The fitted relationships described in this paper provide some of the crucial data that are lacking if mathematical models of schistosomes and water temperature are to be fitted to additional species of snail, and if smaller-scale site-specific predictions are to be made about the potential effects of climate change on schistosomiasis. Further experiments are needed to determine the effects of water temperature on *B. sudanica* egg and juvenile development and mortality rates. In conjuncture with our work, these data would enable the temperature at which the intrinsic rate of increase of *B. sudanica* populations is highest to be estimated, and would allow accurate and complete model parameterisation to *B. sudanica*.

In contrast to the results for snail mortality and fecundity, no clear relationship between water temperature and snail growth could be observed. Although there was a highly significant relationship between water temperature and growth for small and medium snails, and a quadratic relationship gave a significantly better fit to the data than a linear relationship, variation in temperature only explained 16% and 11% of the overall variation respectively. For large snails, there was no evidence of any relationship between water temperature and growth. For these reasons, it is important not to place too much confidence in the estimated temperatures for optimum growth. The high level of variation in snail shell growth suggests that further experiments with many more individual snails, and multiple aquariums at each temperature, may be beneficial in elucidating the true nature of any relationship between water temperature and *B. sudanica* growth.

Similar laboratory experiments have been conducted with three other species of *Biomphalaria* snail: *B. pfeifferi* [24-28], *B. alexandrina* [29], and *B. glabrata* [30,31]. Making direct comparisons between the absolute mortality and fecundity rates found by different studies is problematic, as the observed differences may be due to differences in the experimental set-ups only, and may not reflect genuine variation between the snail species in their natural environments. It is more informative to compare estimated optimum temperatures or temperature ranges. These comparisons suggest two main differences between *B. sudanica* and the three other *Biomphalaria* species. The first is that *B. sudanica* appear to have a distinct peak temperature for survival, while the other species have a wider plateau of highly suitable temperatures. The second is that the optimum temperature for *B. sudanica* egg production is low at only 22°C, compared to 24-27°C for the other three species [24-31].

Due to logistical reasons, the laboratory based snail experiments were conducted in two parts. In the first, snails were kept at temperatures of approximately 15°C, 19°C, 23°C, 27°C, and 31°C. In the second, the snails were kept at 13°C, 17°C, 21°C, 25°C, and 29°C. Care was taken to keep any avoidable differences between the two experiments, other than temperature, at a minimum. Nevertheless, the results for snail mortality and fecundity suggest that there were slight differences between the two experiments. These differences were not large however, and will have little effect on any conclusions drawn from the study, or on the utility of the results for future mathematical modelling work. Two factors that may have contributed to the observed differences are different conditions in Lake Albert during the snails’ juvenile development, and differences in the chemical composition of the water used in the two sets of experiments.

### Effect of population density on *B. sudanica* and *B. stanleyi* population dynamics

Although a number of previous studies have investigated the effects of high snail densities on *Biomphalaria* population dynamics in an artificial laboratory setting [15-18], to the best of our knowledge, this study is the first to investigate the effects in a more natural setting. Our results suggest that *B. sudanica* and *B. stanleyi* snails adopt different strategies to optimise their lifetime fecundity in response to the stresses caused by above optimum snail densities. With a four-fold increase in snail densities, *B. sudanica* fecundity fell by 76%, but there was no evidence of any increase in mortality rates. With the same increase in snail densities, *B. stanleyi* fertility rates decreased by a similar amount (71%), but this was also accompanied by a doubling of mortality rates.

Previous laboratory experiments suggest that the strategy employed by *B. sudanica* is more typical for *Biomphalaria* species. In a laboratory, *B. pfeifferi* produced fewer eggs at higher population densities, but there was no increase in mortality rates or the length of time before juvenile snails started laying eggs [18]. Similar experiments with *B. glabrata* [15,17] and *B. alexandrina* snails [16] also showed reductions in fecundity with increases in snail densities above a threshold level, but no significant effect on mortality rates. The much greater effect of high population densities on fecundity rates compared to mortality rates means that *Biomphalaria* populations are likely to have an inherent tendency towards large fluctuations in numbers. In many habitats, these will coincide with and be driven by seasonal changes in the abiotic properties of the water body, however large fluctuations in population numbers may occur even in the absence of such seasonal changes.

The main limitation of our study is that snail species were identified using morphological methods only. Plam *et al.* compared the morphological and molecular identification of *Biomphalaria* snails from Lake Albert [13]. They found that *B. sudanica* could be clearly differentiated from other species found in the lake using morphological methods only, due to their uniquely low aperture and slowing coiling whorls. The identification of *B. stanleyi* was less clear however, with some *B. stanleyi* being morphologically similar to some *B. choanomphala* and *B. pfeifferi*. It is therefore possible that not all of the snails identified as *B. stanleyi* were *B. stanleyi*. The strong trends towards increasing mortality and decreasing fecundity with increasing snail densities suggest that this had only a minor effect on the results however, either due to only a minority of snails being wrongly identified, and/or due to increasing densities having a similar effect on all three species.

## Conclusions

In conclusion, we have provided valuable information that highlights the sensitivity of snail species capable of hosting *S. mansoni* infections to abiotic conditions within the water habitat. Wherever there is temporal variation in water temperatures, the snail population dynamics may exhibit seasonal changes. Our results, in combination with observations from both lake [14] and pond [32] settings, also indicate that snail populations may exhibit large variations in numbers, even with little or no apparent change in environmental conditions. Monitoring snail populations will therefore yield some insights into changes in human infection risk, but may not be a suitable proxy in all settings unless conducted over long time periods. We recommend that, given the realities of climate change, empirical research into snail ecology is amplified to provide substantial information for informing models that can be used to predict future changes in risk to humans.

## Additional file

Additional file 1:
**Empirical data and fitted relationships.** Empirical data and fitted relationship showing the effects of water temperature on B. sudanica fecundity, mortality and growth rates.
