# Supplementary material for: Effect of water temperature and population density on the population dynamics of Schistosoma mansoni intermediate host snails
Source: Parasit Vectors. 2014 Nov 12;7:503. doi: 10.1186/s13071-014-0503-9 (PMC4234839; doi:10.1186/s13071-014-0503-9)
Supplement: Additional file 1: — Empirical data and fitted relationships. Empirical data and fitted relationship showing the effects of water temperature on B. sudanica fecundity, mortality and growth rates. [file 13071_2014_503_MOESM1_ESM.docx]

| Mean water temperature | Mean eggs/  snail/week (95% CI) | Mean egg masses/  snail/week (95% CI) | Mean eggs/  egg mass (95% CI) |
| --- | --- | --- | --- |
| 13.4°C | 0.82 (0.70-0.96) | 0.16 (0.11-0.22) | 5.38 (4.10-6.65) |
| 15.7°C | 26.02 (18.92-35.78) | 1.85 (0.88-3.87) | 14.1 (13.16-15.04) |
| 16.7°C | 45.38 (33.01-62.39) | 3.45 (1.65-7.20) | 13.75 (12.42-15.08) |
| 18.9°C | 59.69 (43.45-81.99) | 3.44 (1.66-7.16) | 17.36 (16.28-18.45) |
| 20.9°C | 60.47 (44.00-83.11) | 3.97 (1.91-8.29) | 14.92 (13.17-16.68) |
| 22.8°C | 49.00 (35.66-67.33) | 2.87 (1.38-5.98) | 16.42 (15.32-17.52) |
| 26.7°C | 49.27 (35.84-67.73) | 3.87 (1.85-8.07) | 11.04 (9.56-12.53) |
| 28.3°C | 35.80 (26.04-49.21) | 2.23 (1.07-4.66) | 14.68 (13.55-15.81) |
| 29.5°C | 20.74 (15.04-28.58) | 1.93 (0.91-4.07) | 10.88 (8.46-13.30) |
| 32.0°C | 4.11 (2.93-5.78) | 0.51 (0.23-1.15) | 7.36 (5.01-9.71) |
| Fitted relationship | $k*\left( \frac{T_{w}+\theta}{\lambda} \right)^{\alpha-1}* \left( 1- \left. \frac{T_{w}+\theta}{\lambda} \right. \right)^{\alpha-1}$ | | |
|  | k=243.45  α=1.88  β=2.18  θ=-13.4  λ=19.22 | k=9.85  α=1.63  β=1.79  θ=-13.39  λ=19.07 | k=26.71  α=1.27  β=1.53  θ=-13.35  λ=20.66 |

**Table S1. Empirical data and fitted relationships on the effect of water temperature on snail fecundity.** Model fitting is described in the methods section of the paper. T_w_ = water temperature. Other fitted parameters have no direct biological meaning.

|  | Mortality rate (per week) | 95% CI |
| --- | --- | --- |
| 13.4°C | 0.064 | 0.027 - 0.101 |
| 15.7°C | 0.112 | 0.074 - 0.149 |
| 16.7°C | 0.036 | 0.010 - 0.062 |
| 18.9°C | 0.048 | 0.027 - 0.070 |
| 20.9°C | 0.011 | 0.000 - 0.026 |
| 22.8°C | 0.061 | 0.032 - 0.090 |
| 26.7°C | 0.050 | 0.018 - 0.081 |
| 28.3°C | 0.146 | 0.104 - 0.189 |
| 29.5°C | 0.243 | 0.175 - 0.312 |
| 32.0°C | 0.347 | 0.253 - 0.440 |
| Fitted relationship | *a*$T_{w}^{2}$ *+ b*$T_{w}$*+ c*  a = 0.0021  b = -0.084  c = 0.86 | |

**Table S2. Empirical data and fitted relationship on the effect of water temperature on snail mortality.** Model fitting is described in the methods section of the paper. T_w_ = water temperature. Other fitted parameters have no direct biological meaning.

|  | Mean growth in shell diameter (95% CI) (mm per fortnight) | | |
| --- | --- | --- | --- |
|  | **Small snails** | **Medium snails** | **Large snails** |
| 13.4°C | 0.32 (0.19 - 0.45) | 0.20 (0.17 - 0.23) | 0.21 (0.12 - 0.29) |
| 15.7°C | 0.61 (0.45 - 0.77) | 0.59 (0.48 - 0.70) | 0.68 (0.52 - 0.84) |
| 16.7°C | 0.76 (0.57 - 0.95) | 0.71 (0.64 - 0.78) | 0.56 (0.44 - 0.67) |
| 18.9°C | 0.88 (0.71 - 1.05) | 0.63 (0.54 - 0.72) | 0.60 (0.53 - 0.67) |
| 20.9°C | 1.93 (1.67 - 2.19) | 1.19 (1.07 - 1.32) | 0.70 (0.60 - 0.80) |
| 22.8°C | 1.66 (1.26 - 2.07) | 0.85 (0.71 - 0.99) | 0.70 (0.59 - 0.80) |
| 26.7°C | 2.19 (1.89 - 2.49) | 0.94 (0.80 - 1.07) | 0.70 (0.57 - 0.83) |
| 28.3°C | 1.23 (0.94 - 1.52) | 0.79 (0.69 - 0.89) | 0.48 (0.38 - 0.57) |
| 29.5°C | 0.74 (0.48 - 1.00) | 0.56 (0.46 - 0.65) | 0.54 (0.41 - 0.67) |
| 32.0°C | 1.36 (0.85 - 1.87) | 0.88 (0.66 - 1.10) | 0.81 (0.47 - 1.15) |
| Fitted relationship | *a*$T_{w}^{2}$ *+ b*$T_{w}$*+ c*  a = -0.016  b = 0.75  c = -6.98 | *a*$T_{w}^{2}$ *+ b*$T_{w}$*+ c*  a = -0.0059  b = 0.27  c = -2.25 | $aT_{w}$*+ b*  a = 0.012  b = 0.33 |

**Table S3. Empirical data and fitted relationship on the effect of water temperature on snail growth.** Model fitting is described in the methods section of the paper. T_w_ = water temperature. Other fitted parameters have no direct biological meaning.
